# Development of Freeze-Dried Hyaluronic Acid Sheets for Healing Oral Mucositis: Influence of Hyaluronic Acid Molecular Weight and Nicotinamide Mononucleotide Loading on Healing Efficacy

**DOI:** 10.3390/jfb17030137

**Published:** 2026-03-10

**Authors:** Akiko Tanaka, Takanobu Takata, Hidemasa Katsumi, Yasuhisa Sawai, Hiroyuki Nakano, Chika Yoneto, Kunio Yoneto, Tomoyuki Furubayashi, Toshiyasu Sakane

**Affiliations:** 1 Laboratory of Pharmaceutical Technology, Kobe Pharmaceutical University, Kobe 658-8558, Japan; 2 Division of Molecular and Genetic Biology, Department of Life Science, Medical Research Institute, Kanazawa Medical University, Uchinada 920-0293, Japan; 3 Department of Pharmacy, Kanazawa Medical University Hospital, Uchinada 920-0293, Japan; 4 Department of Pharmaceutics, Graduate School of Pharmacy, Showa Medical University, Shinagawa-ku, Tokyo 142-8555, Japan; 5 Department of Oral and Maxillofacial Surgery, Kanazawa Medical University, Uchinada 920-0293, Japan; 6 Ritapharma, Co., Ltd., Shimogyo-ku, Kyoto 600-8813, Japan

**Keywords:** hyaluronic acid, oral mucositis, nicotinamide mononucleotide, drug delivery system

## Abstract

Oral mucositis frequently develops during radiotherapy or chemotherapy for head and neck cancer and is characterized by severe pain and impaired eating and speech. It was previously demonstrated that freeze-dried hyaluronic acid (HA) sheets effectively promote the healing of oral mucosal ulcers. This study aimed to optimize the HA sheet formulation by evaluating the effects of HA molecular weight and nicotinamide mononucleotide (NMN) loading on therapeutic efficacy. HA sheets were prepared using HA with four different molecular weights (50, 350, 800, and 2000 kDa), and their therapeutic effects were evaluated in an animal oral ulcer model using 6-week-old male Syrian hamsters. Among the formulations tested, the 800 kDa HA sheet exhibited the greatest healing efficacy, and it showed an excellent balance between buccal retention and the sustained release of NMN for the treatment of oral mucositis. In vitro cytotoxicity assays confirmed that HA, with or without NMN, was non-toxic and suitable for local applications. These findings indicate that HA sheets, particularly those composed of 800 kDa HA, may represent a promising and biocompatible mucoadhesive material for the delivery of NMN and the local treatment of oral mucositis associated with head and neck cancer.

## 1. Introduction

Patients with head and neck cancer frequently develop oral mucositis, a severe adverse effect of radiotherapy or chemoradiotherapy [1,2,3,4]. Oral mucositis is characterized by painful ulcerative lesions, impaired oral intake, and reduced quality of life [5]. Severe cases often require opioid analgesics, topical anesthetics, or mucosal coating agents for symptom management [6,7]. These interventions can alleviate pain; however, they do not accelerate healing or prevent recurrence, thereby imposing a substantial physical and emotional burden on patients. Despite the clinical significance of oral mucositis, no effective preventive or curative therapy has been established.

Hyaluronic acid (HA) is a naturally occurring glycosaminoglycan [8,9,10,11] with excellent biocompatibility and biodegradability [9,11,12] and plays key roles in tissue hydration [13], extracellular matrix maintenance [11,13], and wound healing [14,15,16]. To exploit these properties, HA is commonly formulated as a topical gel for the treatment of mucosal lesions. However, conventional HA-based gels often require pH adjustment or chemical modifications, such as covalent crosslinking, to achieve gelation [17,18]. These processes may raise concerns regarding mucosal compatibility and potential tissue irritation when applied to damaged oral mucosa. To overcome the limitations of pH-dependent gel formulations, alternative dosage forms that do not require chemical modification and provide physical protection to the lesion are desirable. Thin films and membranes are widely used in industrial and medical fields as protective layers, functional barriers, and structural components in devices such as filters, surgical instruments, and sensors [19]. Based on these concepts, freeze-dried HA sheets were developed as bioadhesive membranes for oral mucosal applications [20,21]. Importantly, these HA sheets are fabricated without pH adjustment or chemical additives and undergo gelation upon contact with saliva after application. They adhere to mucosal surfaces for prolonged periods, offering a significant advantage over short-acting rinses and gels. They exhibit intrinsic wound-healing effects, are biodegradable, and can incorporate water-soluble drugs for localized delivery. In a previous study, we demonstrated that HA sheets alleviated acetic acid-induced oral mucositis in hamster models, confirming their in vivo wound-healing efficacy [20]. In addition, the pilocarpine-loaded HA sheets demonstrated sustained drug release and therapeutic benefits in evaluation studies using hamsters and rats [21], highlighting the versatility of HA sheets as multifunctional platforms for oral mucosal therapy.

Importantly, the physicochemical properties of HA, particularly its molecular weight, strongly influence its mucosal retention, wound-healing activity, and drug release behavior. However, the optimal molecular weight of HA for oral mucosal therapy has not yet been clearly established. Therefore, the optimal molecular weight of HA is essential to maximize the therapeutic potential of HA-based delivery systems. To systematically evaluate the influence of molecular weight, HA samples were selected to cover a broad molecular weight range from low to high molecular weights (50–2000 kDa). In this study, HA sheets with four different molecular weights (50, 350, 800, and 2000 kDa) were prepared, and the optimal formulation was identified based on in vivo performance. The therapeutic effect of incorporating nicotinamide mononucleotide (NMN), a molecule reported to enhance cellular energy metabolism, promote DNA repair, reduce oxidative stress, and preserve tissue integrity [22,23,24], into an optimized HA sheet for the treatment of oral mucositis was further evaluated. Given that oral mucositis involves significant oxidative damage and impaired regeneration, this strategy aims to establish a novel locally delivered therapy that combines the intrinsic wound-healing properties of HA with the biological activities of NMN, thereby evaluating the synergistic therapeutic potential of the optimized NMN-HA sheet as a promising alternative to conventional symptomatic treatments.

## 2. Materials and Methods

### 2.1. Materials

Acetic acid (99.7%) was purchased from FUJIFILM Wako Pure Chemical Corporation (Osaka, Japan). Fluorescein isothiocyanate–dextran (FD4) was purchased from Sigma-Aldrich (St. Louis, MO, USA). Ammonium formate and methanol (HPLC grade) were purchased from Nacalai Tesque, Inc. (Kyoto, Japan). Dulbecco’s Modified Eagle’s Medium (DMEM)/Ham’s F-12 and Triton X-100 were also purchased from Nacalai Tesque, Inc. (Kyoto, Japan). Fetal bovine serum and penicillin–streptomycin were purchased from Gibco (Thermo Fisher Scientific, Waltham, MA, USA). HA with molecular weights of 50, 350, and 800 kDa was supplied by Kewpie Corporation (Tokyo, Japan). HA with a molecular weight of 2000 kDa was obtained from Shinei Chemical Co., Ltd. (Osaka, Japan). NMN was purchased from IM System Co., Ltd. (Kyoto, Japan).

### 2.2. Animals

Six-week-old male Syrian hamsters were purchased from Japan SLC Inc. (Shizuoka, Japan). All animal experiments were conducted according to the principles and procedures outlined in the National Institutes of Health Guide for the Care and Use of Laboratory Animals (NIH publication #85-23). All animal experiments were approved by the Animal Experiment Committee of the Kobe Pharmaceutical University (#2025-032).

### 2.3. Preparation of Freeze-Dried HA Sheet, NMN-Containing HA Sheet (NMN-HA Sheet), and Fluorescein Isothiocyanate–Dextran 4 (FD4)-Containing HA Sheet (FD4-HA Sheet)

HA sheets were prepared according to previously reported methods [20,21]. HA with molecular weights of 50, 350, 800, and 2000 kDa (60 mg each) were briefly dissolved in 6 g of distilled water. A mixture of 800 kDa HA (60 mg) and NMN (60 mg) was dissolved in 6 g of distilled water. NMN was incorporated at a 1:1 (*w*/*w*) ratio relative to HA based on formulation design considerations to balance dissolution properties and drug loading. For the retention study using a non-absorbable marker, fluorescein isothiocyanate–dextran 4-containing HA (FD4-HA) sheets were prepared by dissolving 60 mg of HA (350, 800, and 2000 kDa) and 2 mg of fluorescein isothiocyanate–dextran 4 (FD4, MW 4,000) in 6 g of distilled water.

Each solution was transferred to a dish (radius 3.4 cm) and lyophilized using an FDU-2200 freeze dryer (EYELA, Tokyo, Japan) at 30 Pa for two days.

### 2.4. Scanning Electron Microscopy Observation of HA Sheets

The surface morphologies of the HA and NMN-HA sheet were observed using a scanning electron microscope (FlexSEM 1000, Hitachi, Tokyo, Japan) after osmium coating (HPC-100, Vacuum Device, Ibaragi, Japan) using the backscattered electron mode at an acceleration voltage of 15 kV under high-vacuum conditions.

### 2.5. Preparation of an Oral Mucositis Model in Hamsters

Hamsters were anesthetized via intraperitoneal administration of medetomidine (0.3 mg/kg), midazolam (2.0 mg/kg), and butorphanol (2.5 mg/kg). A filter paper (8 mm × 8 mm) soaked in 70% acetic acid was applied to the buccal mucosa for 3 min and then air-dried for 4 min to induce oral mucositis [20]. After induction of oral mucositis, animals were randomly assigned to the control and treatment groups. Blinding was not performed in this study, and investigators were aware of the group allocation during the allocation, conduct of the experiment, outcome assessment, and data analysis.

### 2.6. Therapeutic Effects of HA Sheets Prepared with Different Molecular Weights on Oral Mucositis

The therapeutic effects of HA sheets prepared with different molecular weights were evaluated in hamsters with mucositis. Two days after acetic acid application (designated as day 1), HA sheets made of four HA molecular weights (50, 350, 800, and 2000 kDa) were applied to the inflamed buccal mucosa. Five groups were compared: four treatment groups receiving the respective HA sheets and a non-treatment control group without HA sheet application. The sheets were applied daily from day 1 to 4, and the lesion area was measured daily until day 5 in all groups. The lesion area was calculated by measuring the vertical and horizontal dimensions of the inflamed site with a ruler and multiplying them.

### 2.7. Retention of HA Sheets on the Buccal Mucosa

HA sheets containing 2 mg of the non-absorbable marker FD4 and prepared using three different HA molecular weights (350, 800, and 2000 kDa) were cut into quarters and applied to the buccal mucosa of hamsters. At 1, 3, and 6 h after application, the remaining HA sheets were collected, dissolved in water, and the fluorescence intensity was measured using a spectrofluorometer (excitation/emission: 485/538 nm; FLUOROSCAN ASCENT FL, Thermo Fisher Scientific Inc., Waltham, MA, USA). The retention rate of each HA sheet was calculated by dividing the remaining amount by the initial amount, which was determined based on the proportion of the applied sheet relative to the total weight of the original sheet.

### 2.8. Therapeutic Effects of NMN-HA Sheets on Oral Mucositis

To assess the effect of NMN, HA sheets prepared from 800 kDa HA were applied to the buccal mucosa of hamsters with oral mucositis, with or without NMN. Three groups were compared: HA sheet (800 kDa), NMN-HA sheet (800 kDa), and untreated control. The sheets were applied daily from days 1 to 4, and the lesion area was measured daily from days 1 to 5, as described for the HA molecular weight comparison study.

### 2.9. In Vitro Release of NMN from NMN-HA Sheets

The in vitro release of NMN from the NMN-HA sheets was evaluated using Franz-type diffusion cells. NMN-HA sheets prepared from 800 or 2000 kDa HA, containing 60 mg of NMN per sheet, were compared with NMN dissolved in water (30 mg/mL, 250 µL on the donor side). Donor-side NMN-HA sheets were cut to fit the donor compartment, and phosphate-buffered saline (PBS) was added so that each sheet corresponded to 2 mL. The initial amount of NMN in each piece was calculated based on the proportion of the sheet’s weight relative to the total sheet. The receptor compartment contained 3 mL of PBS (pH 7.4) and was maintained at 37 °C with continuous magnetic stirring. At 15, 30, 60, 120, 180, 240, and 360 min, 300 µL aliquots were collected from the receptor solution and immediately replaced with an equal volume of fresh PBS. NMN concentrations in the receptor solution were determined by HPLC using a COSMOSIL 5C8-MS column (4.6 mm × 150 mm, Nacalai Tesque, Inc., Kyoto, Japan). The mobile phase was 20 mM ammonium formate, with an injection volume of 20 µL and detection at 260 nm. Samples were appropriately diluted with PBS to ensure that NMN concentrations fell within the linear range of the calibration curve (0.5–10 µg/mL). Quantification was performed using an external calibration curve with good linearity (R^2^ > 0.99). The cumulative release of NMN was calculated relative to the initial NMN content of the NMN-HA sheet.

### 2.10. Cytotoxicity Evaluation of HA and NMN

The cytotoxicity of HA and NMN was evaluated in vitro using colorimetric cell viability assays. HeLa cells were used as representative cell lines for cytotoxicity screening.

HeLa cells were cultured in Dulbecco’s Modified Eagle’s Medium (DMEM)/Ham’s F-12 supplemented with 10% fetal bovine serum and 1% penicillin–streptomycin at 37 °C in a humidified atmosphere containing 5% CO_2_. Cells were seeded into 96-well plates at a density of 1.0 × 10^4^ cells per well and allowed to adhere for 24 h prior to treatment.

HA solutions were prepared from 800 kDa HA at concentrations of 0.001, 0.01, and 0.1 mg/mL. NMN solutions were prepared at concentrations of 0.001, 0.01, and 0.1 mM. Additionally, a combined formulation of HA (0.1 mg/mL) and NMN (0.1 mM) was prepared to evaluate the cytotoxicity of the co-administered system. Each test solution (100 µL) was added to the cells, followed by incubation for 24 h.

Cells cultured in fresh medium served as negative controls, whereas cells treated with medium containing 0.1% Triton X-100 served as positive controls. After treatment, 10 µL of CCK-8 reagent was added to each well, and the plates were incubated for an additional 1 h according to the manufacturer’s instructions (CCK-8; Dojindo Laboratories, Kumamoto, Japan). The absorbance was measured at 450 nm using a microplate reader, and cell viability was expressed as a percentage of the negative control.

### 2.11. Statistical Analysis

All quantitative data are expressed as the mean ± standard error (SE). Statistical analyses were performed using StatView version 5.0 (SAS Institute Inc., Cary, NC, USA). For time-course experiments, comparisons among treatment groups were performed independently at each time point using one-way analysis of variance (ANOVA), followed by the Tukey–Kramer multiple comparison test. A *p* value of <0.05 was considered statistically significant.

## 3. Results and Discussion

### 3.1. SEM Observation of HA and NMN-HA Sheets

**Figure 1:**
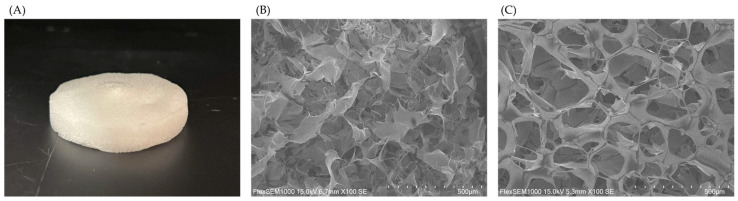
Photograph of HA sheet (**A**) and SEM images of HA sheet (**B**) and NMN-HA sheet (**C**).

The HA sheets prepared in this study exhibited uniform surfaces, indicating a homogeneous matrix. In contrast, the incorporation of NMN resulted in a more porous structure, as shown in the SEM images (Figure 1). This increase in porosity may facilitate water uptake into the matrix, thereby potentially enhancing NMN release.

### 3.2. Therapeutic Effects of HA Sheets with Different Molecular Weights

**Figure 2:**
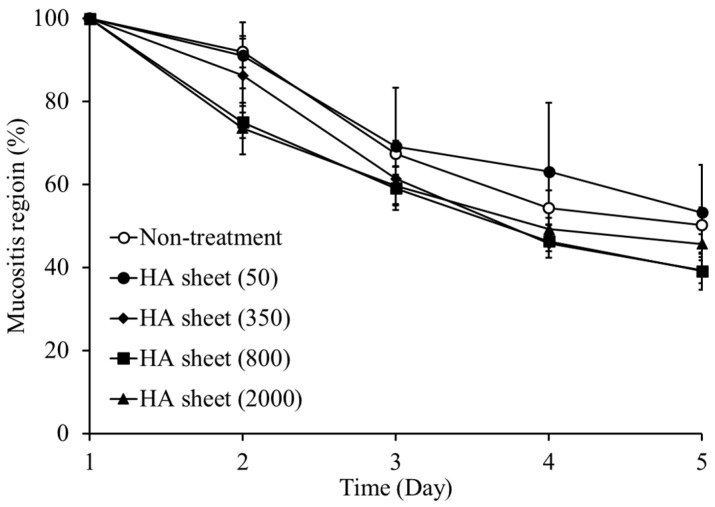
Effect of HA molecular weight on the healing of oral mucositis. The therapeutic efficacy of HA sheets with different molecular weights was evaluated by measuring the size of oral mucositis lesions over time. Data are presented as mean ± standard error (SE). Symbols indicate treatment groups: ○ non-treatment (n = 6), ● 50 kDa (n = 4), ♦ 350 kDa (n = 4), ■ 800 kDa (n = 5), ▲ 2000 kDa (n = 6).

To determine the optimal molecular weight of HA for therapeutic efficacy, HA sheets with four different molecular weights (50, 350, 800, and 2000 kDa) were evaluated using a hamster model of oral mucositis (Figure 2). Although the acetic acid-induced oral mucositis model is widely used to evaluate mucosal injury and repair [25], it may not fully reflect the complex pathogenesis of clinical oral mucositis associated with chemotherapy or radiotherapy. Nevertheless, this model reproduces several key pathological features of oral mucositis and has been widely used for evaluating therapeutic interventions targeting mucosal healing. Therefore, it remains a useful experimental platform for the comparative evaluation of formulation performance.

In this experimental framework, two days after acetic acid application was designated as day 1, and lesion areas were monitored from days 2 to 5. For clarity, particular attention was focused on day 2, corresponding to the early inflammatory phase, and day 5, representing the later stages of healing.

In the early phase (day 2), a clear numerical trend was observed: higher-molecular-weight HA formulations exhibited greater suppression of mucosal lesions. The lesion area in the non-treatment group remained at 92.0 ± 3.8%. In comparison, the 800 kDa group showed a reduced lesion area of 75.0 ± 3.9%, corresponding to an 18.5% reduction relative to the non-treatment group. Notably, the 2000 kDa group showed a further decrease to 73.5 ± 6.2%, representing a 20.1% reduction. These findings indicate a strong early anti-inflammatory tendency in high-molecular-weight HA sheets, with the 2000 kDa group showing the greatest numerical effect during the initial phase.

However, by day 5, the efficacy profile shifted. The 800 kDa group maintained the smallest lesion area (39.2 ± 4.5%), closely followed by the 350 kDa group (39.3 ± 3.1%). In contrast, the 2000 kDa group exhibited a smaller reduction in lesion area (45.7 ± 2.4%) compared with the 350 and 800 kDa groups. Relative to the non-treatment group (50.2 ± 4.3%), the 800 kDa group achieved a 21.9% reduction in the lesion area, representing the most pronounced numerical improvement at this stage.

The effects of HA on cell proliferation, migration, and inflammation have been reported to depend on its molecular weight [26,27,28]. In the present study, HA sheets prepared from 800 and 2000 kDa HA showed a tendency toward earlier suppression of inflammation. However, the slightly attenuated efficacy of the 2000 kDa HA observed at day 5 indicates that excessively high molecular weight is not necessarily optimal during the later stages of the healing process. This reduction cannot be explained solely by differences in the physical coverage of the mucosal surface. It may instead reflect limitations in local diffusion associated with higher viscosity, as well as altered degradation behavior under inflammatory conditions. In inflamed tissues, increased hyaluronidase activity and oxidative stress accelerate HA depolymerization, particularly in high-molecular-weight species [29,30]. Although such HA may initially form a robust physical coating on the mucosal surface, rapid depolymerization under inflammatory conditions may compromise the persistence of its anti-inflammatory and biological activities, rather than its physical presence alone.

Taken together, these findings indicate that the therapeutic performance of HA sheets is governed by an optimal balance between biological activity, diffusibility, and stability, rather than molecular weight alone. Among the formulations tested, HA, with a molecular weight of approximately 800 kDa, achieved the most favorable balance, resulting in the most sustained therapeutic efficacy. Consequently, this formulation was selected for subsequent experiments. To further elucidate the contribution of mucosal residence to this optimal performance, the retention behavior of the selected HA sheets was evaluated in subsequent experiments.

### 3.3. Retention of HA Sheets on the Buccal Mucosa

**Figure 3:**
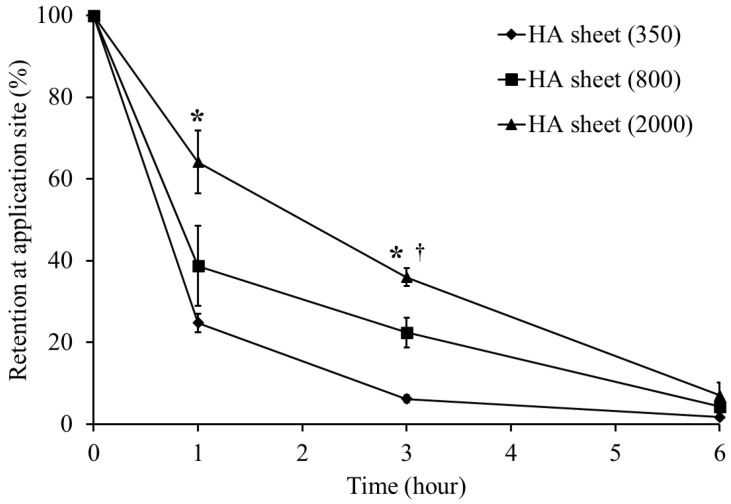
Retention of HA sheets on the buccal mucosa. The retention behavior of HA sheets with different molecular weights on the buccal mucosa was evaluated over time. Data are presented as mean ± standard error (SE). Symbols indicate HA molecular weights: ♦ 350 kDa (n = 5), ■ 800 kDa (3 h: n = 4; other time points: n = 5), ▲ 2000 kDa (1 h: n = 4; 3 h: n = 4; other time points: n = 5). * *p* < 0.05: significantly different from the HA sheet (350 kDa) group. † *p* < 0.05: significantly different from the HA sheet (800 kDa) group.

Given that HA sheets dissolve in saliva and their viscosity depends on molecular weight [31,32], their retention in the buccal mucosa was evaluated (Figure 3). One hour after application, the remaining fractions of the 2000, 800, and 350 kDa groups were 64.1, 38.7, and 24.8%, respectively, confirming that a higher molecular weight increases viscosity and thereby enhances mucosal retention. These results indicate that these molecular weight-dependent biological properties and retention characteristics likely contributed to the enhanced suppression of inflammation observed in the higher-molecular-weight groups (800 and 2000 kDa) in the early phase (day 2). However, after 6 h, the residual amounts decreased in all groups, and by 24 h, only the 2000 kDa group showed slight retention (4.6%). Importantly, despite its superior retention, the 2000 kDa formulation did not exhibit the greatest therapeutic efficacy at the later stage of healing (day 5). These findings also indicate that mucosal retention alone does not fully account for the differences in therapeutic efficacy. Excessively high viscosity may not necessarily be optimal for sustained therapeutic outcomes, as it may limit the local diffusion of HA across the mucosal surface and alter its degradation behavior under inflammatory conditions. Taken together, these results highlight the importance of achieving an appropriate balance between mucosal retention and effective local bioavailability, leading to the selection of the 800 kDa HA sheet for subsequent experiments.

### 3.4. Therapeutic Effects of NMN-Containing HA Sheets on Oral Mucositis

**Figure 4:**
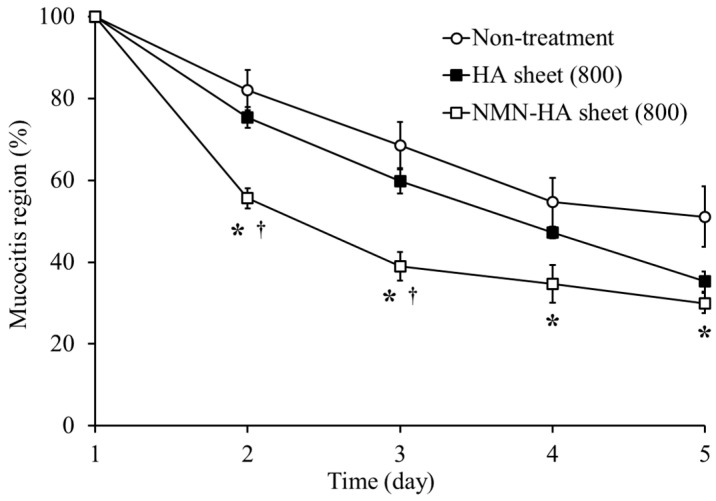
Therapeutic effect of NMN-HA sheets on oral mucositis. The therapeutic efficacy of HA sheets with or without nicotinamide mononucleotide (NMN) was evaluated by measuring the size of oral mucositis lesions over time. Data are presented as mean ± standard error (SE). Symbols indicate treatment groups: ○ non-treatment (n = 4), ■ HA sheet (800 kDa) (n = 6), □ NMN-HA (800 kDa) sheet (n = 5). * *p* < 0.05: significantly different from the non-treatment group. † *p* < 0.05: significantly different from the HA sheet group.

Based on the findings described above, which identified the 800 kDa HA sheet as the optimal formulation, NMN was incorporated into this sheet to evaluate its additional therapeutic benefits in a hamster model of oral mucositis (Figure 4).

On day 2, lesion areas were 82.0 ± 4.9% in the non-treatment group, 75.4 ± 2.6% in the HA sheet group, and 55.6 ± 2.5% in the NMN-HA sheet group. Compared to the non-treatment group, the NMN-HA sheet group achieved a substantial reduction in the lesion area of approximately 32.2%. Furthermore, compared with the HA sheet group, the addition of NMN resulted in a 26.3% reduction in the lesion size on day 2. By day 3, lesion areas were 68.6 ± 5.7, 59.7 ± 3.0, and 39.0 ± 3.5% in the non-treatment group, the HA sheet group, and the NMN-HA sheet group, respectively, with the NMN-HA sheet group maintaining a 43.1% reduction relative to the non-treatment group. At day 5, the lesion areas reached 51.1 ± 7.4, 35.2 ± 2.5, and 30.0 ± 2.5%, respectively.

The addition of NMN to the 800 kDa HA sheet significantly accelerated the reduction in lesion area, particularly during the early to intermediate phases of mucositis. On days 2, 3, and 4, the NMN-HA sheet group showed statistically significant improvements compared with both the HA sheet and non-treatment groups (*p* < 0.05). By day 5, both the HA sheet and NMN-HA sheet groups continued to exhibit significantly smaller lesion areas than the non-treatment group (*p* < 0.05), demonstrating maintained therapeutic efficacy throughout the observation period.

The therapeutic enhancement conferred by NMN was particularly pronounced during the early phase (days 2 and 3). The marked reduction in the lesion area during these initial days provides direct experimental evidence that NMN incorporation significantly accelerates wound healing compared with HA sheets alone, suggesting that NMN, a small-molecule compound, may effectively permeate the wound site [33] and facilitate the activation of cellular repair mechanisms within the mucosal tissue [22,23,24]. These findings indicate that NMN-HA sheets provide superior therapeutic efficacy by accelerating the healing process through the pharmacological effects of NMN, combined with the intrinsic wound-healing capacity of the 800 kDa HA sheet. However, the detailed biological mechanisms underlying the therapeutic effects of NMN, such as modulation of inflammatory cytokines, oxidative stress, and tissue regeneration, were not directly evaluated in the present study. These investigations are beyond the scope of the current work and will be addressed in future studies. Although the present findings demonstrated the therapeutic potential of NMN-HA sheets in the acetic acid-induced oral mucositis model, further studies using chemotherapy- or radiotherapy-induced models will be required to confirm the clinical applicability of this formulation.

### 3.5. In Vitro Release of NMN from HA Sheets

**Figure 5:**
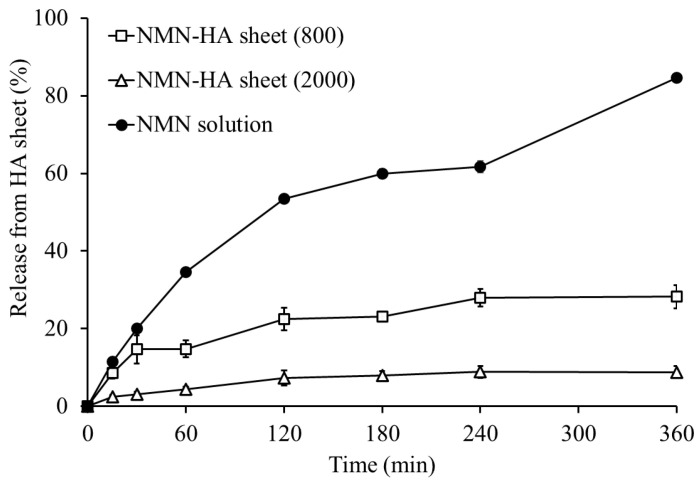
In vitro NMN release from NMN-HA sheets. The in vitro release profiles of nicotinamide mononucleotide (NMN) from HA sheets were evaluated. Data are presented as mean ± standard error (SE). Symbols indicate formulations: □ NMN–HA sheet (800 kDa), △ NMN–HA sheet (2000 kDa), ● NMN solution.

To clarify how the HA sheets function as a delivery system for NMN, the in vitro release profiles of the NMN solution, NMN-HA sheets (800 kDa), and NMN-HA sheets (2000 kDa, used as a reference control) were evaluated (Figure 5). NMN dissolved in water diffused rapidly, with 34.6% released at 1 h, 59.9% at 3 h, and 63.0% at 6 h. In contrast, NMN-HA sheets exhibited markedly lower release at the same time points, demonstrating a clear sustained-release profile. The 2000 kDa group released only 4.5%, 8.0%, and 8.8% at 1, 3, and 6 h, respectively, whereas the 800 kDa group released 14.8%, 23.0%, and 28.2%, respectively. The SEM images of the HA sheets support these observations. The plain HA sheets showed a uniform structure, whereas the NMN-HA sheets exhibited a more porous structure, regardless of the molecular weight of HA. The increased porosity of NMN-HA sheets may facilitate water uptake into the matrix, thereby promoting NMN diffusion and release.

Between the two HA formulations, the 800 kDa group consistently released more NMN than the 2000 kDa group. Specifically, the 800 kDa matrix released approximately 3.3-, 2.9-, and 3.2-fold higher NMN at 1, 3, and 6 h, respectively, compared to the 2000 kDa matrix. This difference likely reflects the lower viscosity and less dense molecular entanglement of the 800 kDa HA matrix, which facilitates the diffusion of NMN. In contrast, the more entangled, diffusion-resistant structure of the 2000 kDa HA appears to trap NMN molecules more effectively, limiting their availability.

Overall, these findings indicate that NMN incorporation into HA sheets provides a sustained-release profile [34] and that the release rate is significantly influenced by HA molecular weight. The release behavior of the 800 kDa HA sheet aligns with its superior in vivo therapeutic effects, as it ensures continuous NMN availability during the early inflammatory phase, when promoting wound healing is most critical.

### 3.6. Cytotoxicity Evaluation of HA and NMN

**Figure 6:**
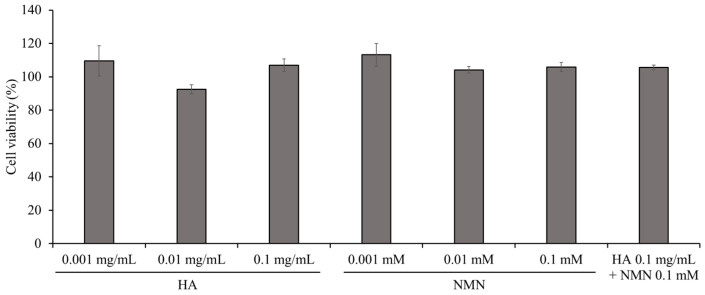
Cytotoxicity of HA sheets and NMN-loaded HA sheets. The cytotoxic effects of HA sheets with or without NMN were evaluated in HeLa cells using a CCK-8 assay. Data are presented as mean ± standard error (SE).

The cytotoxicity of HA (800 kDa), NMN, and their combination was evaluated using the CCK-8 assay (Figure 6). HeLa cells were treated for 24 h with HA solutions (0.001, 0.01, and 0.1 mg/mL), NMN solutions (0.001, 0.01, and 0.1 mM), or a combined formulation of HA (0.1 mg/mL) and NMN (0.1 mM). HeLa cells were used as a representative epithelial cell model because they are widely employed to evaluate cytocompatibility and cellular responses to biomaterials intended for mucosal and epithelial applications [35]. No significant reduction in cell viability was observed at any of the tested HA or NMN concentrations. Cell viability in all treatment groups was comparable to that of the negative control. Importantly, the co-administration of HA and NMN at the highest tested concentrations did not induce detectable cytotoxicity. These results indicated that HA (800 kDa) and NMN are well tolerated under the experimental conditions used in this study. The absence of cytotoxic effects supports the initial safety of the NMN-HA sheets for potential local applications in oral tissues. Although the present study confirmed cytocompatibility using HeLa cells, further biological validation using oral mucosa-derived cells, such as human oral keratinocytes or gingival fibroblasts, will be conducted in future studies to better reflect the intended clinical application.

## 4. Conclusions

This study demonstrated that molecular weight-dependent formulation design influences buccal retention, release of NMN, and wound-healing ability of HA sheets for buccal application. Among various molecular weights of HA, the 800 kDa HA sheet demonstrated superior wound-healing ability, and it showed an excellent balance between buccal retention and the sustained release of NMN for the treatment of oral mucositis. Collectively, these results indicated that the 800 kDa HA sheet is a promising novel topical formulation for the delivery of NMN and the treatment of oral mucositis.

## Data Availability

The original contributions presented in this study are included in the article. Further inquiries can be directed to the corresponding author.
